# Hypothyroidism and Glaucoma in The United States

**DOI:** 10.1371/journal.pone.0133688

**Published:** 2015-07-31

**Authors:** Caitlin Kakigi, Toshimitsu Kasuga, Sophia Y. Wang, Kuldev Singh, Yoshimune Hiratsuka, Akira Murakami, Shan C. Lin

**Affiliations:** 1 Department of Ophthalmology, University of California, San Francisco, San Francisco, California, United States of America; 2 Department of Ophthalmology, Juntendo University School of Medicine, Tokyo, Japan; 3 Department of Ophthalmology, Stanford University, Stanford, California, United States of America; Bascom Palmer Eye Institute, University of Miami School of Medicine;, UNITED STATES

## Abstract

**Purpose:**

To investigate the association between hypothyroidism and glaucomatous disease.

**Methods:**

This cross-sectional study included all subjects above the age of 40 years from two nationwide surveys: the 2008 National Health Interview Survey (NHIS) as well as the 2007 and 2008 National Health and Nutrition Examination Survey (NHANES). The presence or absence of glaucoma, thyroid disease and other demographic and health-related information including comorbidities was ascertained via interview. Blood samples were collected from NHANES subjects and analyzed for thyrotropin (TSH).

**Results:**

A total of 13,599 and 3,839 NHIS and NHANES participants respectively were analyzed to assess for a possible relationship between self-reported glaucoma, and self-reported hypothyroidism as well as self-reported thyroid disease. The unadjusted odds ratio (OR) for NHIS showed a significant association between self-reported glaucoma and self-reported hypothyroidism (OR 1.46, 95% confidence interval [CI] 1.07-1.99). Multivariate logistic regression analysis adjusted for age, gender, race, comorbidities, and health-related behavior, however, showed no association between self-reported glaucoma and hypothyroidism or thyroid disease in both surveys (OR 1.60, 95%CI 0.87-2.95 for NHIS; OR 1.05, 95%CI 0.59-1.88 for NHANES).

**Conclusion:**

A previously reported association between hypothyroidism and glaucomatous disease was not confirmed in two large U.S. health survey populations. While such an association was noted in the univariate analysis for the NHIS survey, such a relationship was not found in the multivariate analysis after adjustment for potential confounding variables.

## Introduction

There have been conflicting reports regarding whether or not hypothyroidism is independently associated with the incidence or prevalence of open angle glaucoma (OAG). Since Hertel’s initial report of two hypothyroidism patients whose intraocular pressure (IOP) was lower after thyroid hormone replacement therapy [1], there have been supportive cases and case series presented by several other groups.[2–8] In contrast, several other reports have shown no association between hypothyroidism and OAG.[9–11] The underlying hypothesis for why hypothyroidism may be an independent risk factor for OAG is based upon the belief that the low metabolic condition caused by this condition results in reduced enzymatic activity that adversely impacts aqueous humor dynamics. A change in the normal cycle of production and degradation of some enzymatic substrates is postulated to result in homeostatic changes that cause increased deposition of hyaluronic acid in the trabecular meshwork resulting in decreased aqueous outflow and a consequent higher IOP.[2,8]

At least five population-based studies have been analyzed for a possible association between thyroid abnormalities and glaucoma.[12–16] Two such studies reported a positive association between hypothyroidism and the likelihood of having OAG [12,15] and others showed an association between thyroid disease and glaucoma.[14,16] One study, however, showed no association between hypothyroidism and OAG.[13]

It remains controversial whether or not hypothyroidism is an independent risk factor for the development or progression of OAG. There have been no population-based studies which have evaluated the possible association between hypothyroidism and OAG based on laboratory confirmation in the form of serum levels of thyroid hormone. The present study evaluates data from two U.S. nationwide population-based surveys—the National Health Interview Survey (NHIS) and the National Health and Nutrition Examination Survey (NHANES)—to further investigate a potential association between hypothyroidism and open angle glaucoma with an arm including laboratory-based confirmation of hypothyroidism.

## Materials and Methods

### Ethics statement

The current study used de-identified publicly available data and was exempt from human subjects review.

### Surveys

The data used for this study were obtained from the 2008 National Health Interview Survey (NHIS) and the National Health and Nutrition Examination Survey (NHANES) for 2007 and 2008. Both NHIS and NHANES provide important health related information regarding the civilian non-institutionalized population of the United States and represent major components of the data collection programs administered by the National Center for Health Statistics (NCHS), which is part of the Centers for Disease Control and Prevention (CDC).

In NHIS, one sample adult from each family is randomly selected and information on each person’s demographics and health are collected via questionnaire. NHANES, by contrast, is a combination of interviews and physical examinations, which include blood tests. NHANES 2007–2008 oversampled persons 60 and older, African Americans, and Hispanics in an effort to accumulate representative health information regarding the non-institutionalized, civilian U.S. population. Details regarding the conduct of NHIS and NHANES including study design, methods and questionnaires are available online.[17,18]

### NHIS

The 2008 NHIS included 29,370 adults (aged 18 years or greater) who were eligible for the sample adult questionnaire of whom 21,781 completed the interviews for a response rate of 74.2%. Our study included 13,599 participants in the 2008 NHIS who were 40 years or older, and completed the adult component of the study. All adult study participants were asked to report whether or not they had ever been told by a physician or other health care professional that they had glaucoma or hypothyroidism. Participants in whom such data could not be ascertained either due to lack of knowledge or unwillingness to share this health care information were excluded from this study. Classification regarding the presence or absence of glaucoma and hypothyroidism was based solely on participants’ self-reported information. Demographic information including age, gender, and race as well as other health information was also collected and used in the analysis.

### NHANES

A total of 10,149 subjects participated in NHANES 2007 and 2008. We included 3,839 participants who were 40 years or older and completed both the interview and examination portions of the NHANES, and excluded all others. All study participants were asked to report whether or not they had ever been told that they had glaucoma or thyroid disease by a physician or other healthcare professional. Blood samples were collected from participants and analyzed for thyrotropin (TSH). Further details on the laboratory analysis protocol for this study can be found on the NHANES website.[19] Participants’ demographic information and other health related data were collected similar to the NHIS portion of this study. The major difference was that NHANES, but not NHIS, had an examination portion of the survey.

### Variable Definitions

The primary outcome variable for both analyses was the self-reported diagnosis of glaucoma. The primary predictor was thyroid disease also based upon self-reporting. In NHIS, the question upon which this self-reporting was based specifically asked whether or not the participant had been told that they had hypothyroidism, while for NHANES the question was posed to determine whether or not the participant had thyroid disease which could be manifest as either hyper- or hypothyroidism. In an effort to account for the undiagnosed cases of hypothyroidism, we performed an additional analysis based on serum TSH levels reported in the NHANES dataset. The secondary predictor for the NHANES was defined as the diagnosis of hypothyroidism confirmed by laboratory values of thyroid-related hormone levels or ascertainment of whether or not study subjects were taking medication for hypothyroidism. Participants who had laboratory-confirmed hyperthyroidism or were taking a prescription medication for hyperthyroidism were excluded from the control group. Information pertaining to drug prescriptions was obtained by interview. Such prescriptions in NHANES study subjects included levothyroxine, methimazole and propylthiouracil. The laboratory-confirmed hypothyroidism patients were further divided into two groups based upon whether or not they were taking medication for hypothyroidism to test the additional hypothesis that levothyroxine may have a protective effect against the development of glaucomatous disease.

### Diagnosis of thyroid condition based on laboratory data for NHANES

For the diagnosis of hypothyroidism we utilized the laboratory reference range for thyroid stimulating hormone (TSH) which was 0.34–5.60 mIU/L based on the manufacturer’s studies using non-parametric analysis of the results measured in 217 human serum samples from apparently healthy male and female subjects with normal thyroid profiles.[19] We considered subjects as ‘hypothyroid’ if their TSH was >5.60 mIU/L or they were taking levothyroxine. Subjects with TSH < 0.34 mIU/L or they were taking prescription for hyperthyroidism were considered to be ‘hyperthyroid’ and excluded from further analysis. Normal thyroid function was defined as the absence of a history of hypo- or hyperthyroidism in those not taking any medication related to a thyroid condition.

### Statistical Analysis

For both the NHIS and the NHANES populations, we compared the distribution of possible confounding factors between participants with and without self-reported glaucoma using design-adjusted Rao-Scott Pearson-type chi-square and Wald tests for categorical and continuous variables, respectively. Multiple logistic regressions were performed to assess the association between those self-reporting glaucoma and thyroid disease, adjusting for socio-demographic factors such as age, gender, race, education, and family income, health related behaviors such as alcohol consumption and tobacco use, comorbid medical conditions, and self-reported general health condition.

In accordance with Motsko et al.[13] and Lin et al.[15], the following medical conditions were considered as potential confounders to be included in the multivariate analysis: diabetes, lipid metabolism disorders, hypertension, cardiovascular disease, cerebrovascular disease, arterial disease, and migraines. Potential confounding comorbidities which were found to be significant at the *P*<0.1 level in multivariate models were included in the final model. The self-reported diagnosis of arterial disease for NHIS subjects, as well as arterial disease and migraine for those participating in NHANES were not available in the dataset and thus this variable was not included in the analysis.

Both NHIS and NHANES adapted a stratified multistage sampling design that required a weighting scheme to provide estimates of disease prevalence in the U.S. population. To account for the stratified and clustered design of the surveys, STATA 12.0 (Stata Statistical Software, College Station, TX) was used for all analyses. P-values less than 0.05 were considered to indicate statistical significance.

## Results

### NHIS

Table 1 summarizes socio-demographic and health characteristics of the 13,599 subjects in the 2008 NHIS that met our inclusion criteria. Among them, 556 had a self-reported diagnosis of glaucoma, and 1,364 had a self-reported diagnosis of hypothyroidism, representing 3.4%, and 9.7% of the overall study population, respectively. Those individuals who reported a diagnosis of hypothyroidism were significantly older, (p<0.0001) more likely to be female (p<0.0001) and non-Hispanic white (p<0.0001) than those who reported not having hypothyroidism. The prevalence of self-reported glaucoma was significantly higher among the self-reported hypothyroidism group compared to those who reported no hypothyroidism (4.7% and 3.3%, respectively. p = 0.0156.) Many other variables also differed significantly between the groups and our final model included the comorbid condition of hypertension.

**Table 1:** Demographics for the National Health Interview Survey (NHIS) based on self-reported hypothyroidism.

	No Hypothyroidism (N = 12,235)		Hypothyroidism (N = 1,364)		
	Mean	Standard Deviation	Mean	Standard Deviation	P value
Age (years)	57.0	12.5	61.5	13.3	<0.0001
Gender, female %	49.4		83.6		<0.0001
Race, %					<0.0001
Hispanic	10.2		7.9		
Non-Hispanic White	73.5		83.9		
Non-Hispanic Black	11.2		5.0		
Non-Hispanic Asian	4.2		2.5		
Other	0.8		0.8		
Education, %					0.1782
<9th Grade	6.9		5.2		
> = 9th Grade, <HS graduate	9.2		8.1		
HS graduate or GED equivalent	29.3		29.8		
Some college	17.1		18.5		
College graduate and byeond	37.6		38.4		
Income, %					<0.0001
<$20,000	22.2		33.4		
$20,000-$44,999	35.1		34.7		
$45,000-$74,999	24.7		20.7		
$75,000 and up	18.0		11.3		
Glaucoma, %	3.3		4.7		0.0156
Diabetes, %	12.0		17.9		<0.0001
High cholesterol, %	38.5		50.3		<0.0001
Hypertension, %	40.9		51.9		<0.0001
Heart attack, %	5.4		8.4		0.0001
Migraine, %	15.5		26.8		<0.0001
Stroke, %	4.1		7.5		<0.0001
BMI	30.2	15.1	33.4	19.3	<0.0001
Smoking, %					0.0124
Current	19.3		15.1		
Former	28.1		30.4		
Never	52.5		54.5		
Alcohol, %					0.0005
Current	61.5		55.3		
Former	18.9		22.9		
Never	19.5		21.8		
General health condition, %					<0.0001
Excellent-Good	82.5		69.0		
Fair	12.9		20.3		
Poor	4.7		10.7		

All means, proportions, and standard erros are weighted estimates of the U.S. population characteristics, taking into account NHIS’s complex sampling design. All P values are unadjusted. P values were calculated using Wald test for continuous variables, and design-adjusted Rao-Scott Pearson chi-square test for categorical variables.

The unadjusted odds ratio (OR) showed significant association between self-reported glaucoma and hypothyroidism (OR 1.46, 95% confidence interval [CI] 1.07–1.99). However, once adjusted for demographic variables such as age, gender, race and other potential confounders, the association was no longer statistically significant. Our final model showed that the OR was 1.60 (95%CI 0.87–2.95) for an association between glaucoma and hypothyroidism. Unadjusted and adjusted odds ratios for hypothyroidism from multiple logistic regression analyses are summarized in Table 2.

**Table 2:** Odd ratios (Ors) of the hypothyroidism for self-reported glaucoma for National Health Interview Survey (NHIS) population.

	Hypothyroidism OR (95% CI)	P-value
Unadjusted	1.46 (1.07–1.99)	0.016
+Age	1.08 (0.77–1.5)	0.652
+Sex	1.03 (0.74–1.43)	0.855
+Race	1.12 (0.81–1.55)	0.503
+Comorbidities	1.09 (0.79–1.51)	0.603
+SES	1.78 (0.92–3.43)	0.086
+HRB	1.72 (0.89–3.31)	0.105
+General health conditions	1.60 (0.87–2.95)	0.133

SES: Socioeconomic status variables included annual household income and education level.

HRB: Health related behaviors include smoking status (current, past, or never), and alcohol intake (current, past, or never).

Comorbidities include hypertension, BMI.

### NHANES

There were 3,839 participants, age 40 years or older from NHANES 2007–2008 who completed both interview and examination portions of the survey, of whom 248 self-reported a diagnosis of glaucoma and 470 self-reported thyroid disease, representing 4.7% and 13.3% of the sampled population, respectively. Similar to the NHIS results, the characteristics of those with and without self-reported thyroid disease differed significantly. Subjects with self-reported thyroid disease were significantly older (p<0.0001) and had more comorbidities. The characteristics of these two groups are summarized in Table 3. For the analysis based on a primary predictor of self-reported thyroid disease, unadjusted odds for a diagnosis of glaucoma in those with hypothyroidism was 1.29 (95% CI 0.81–2.06), and after adjustment for potential demographic and health related confounding factors, the final model showed an OR of 1.05 (95% CI 0.59–1.88). The final model included diabetes as medical comorbidities, but did not include other comorbidities that did not reach significance at P <0.1 in the multivariate model. The ORs for our multivariate logistic regression are summarized in Table 4.

**Table 3:** Demographics for National Health and Nutrition Examination Survey (NHANES) based on self-reported thyroid disease.

	No Thyroid disease (N = 3,369)		Thyroid disease (N = 470)		
	Mean		Mean		P value
Age (years)	56.5	10.9	61.1	61.1	<0.0001
Gender,female %	48.1		83.6		<0.0001
Race, %					<0.0001
Mexican	5.9		4.2		
Other Hispanic	4.3		2.5		
Non-Hispanic white	72.7		84.0		
Non-Hispanic black	11.2		5.7		
Other and multiracial	5.9		3.7		
Education, %					0.0827
<9^th^ Grade	8.9		4.5		
> = 9^th^ Grade, <HS graduate	12.9		11.7		
HS graduate or GED equivalent	26.3		25.8		
Some college	26.4		27.3		
College graduate and beyond	25.5		30.8		
Income, %					0.9158
<$20,000	15.4		15.2		
$20,000-$44,999	27.7		26.3		
$45,000-$74,999	19.7		21.3		
$75,000 and up	33.5		33.7		
>$20,000	3.8		3.4		
Glaucoma, %	4.5		5.8		0.2541
Diabetes, %					0.0364
Yes	11.5		17.5		
Borderline	2.1		2.6		
Hypertension, %	40.7		52.6		0.0014
Stroke, %	4.4		7.2		0.0488
BMI	28.8	5.8	29.3	6.2	0.3150
Smoking, %					0.0919
Never	50.2		53.5		
Current	20.1		14.1		
Past	29.7		32.4		
Alcohol, %					0.1336
0	42.3		49.2		
>0 and <1	42.8		41.3		
≧1 and <2	7.8		6.1		
≧2 and <3	5.1		2.9		
≧4	2.0		0.6		
Genral Health Condition, %					0.0171
Excellent-Good	42.3		32.9		
Fair	38.6		39.4		
Poor	19.1		27.7		

All means, proportions, and standard erros are weighted estimates of the U.S. population characteristics, taking into account NHIS’s complex sampling design.

All P values are unadjusted. P values were calculated using Wald test for continuous variables, and design-adjusted Rao-Scott Pearson chi-square test for categorical variables.

**Table 4:** Odds ratios (Ors) of the self-reported thyroid disease for self-reported glaucoma for National Health and Nutrition Examination Survey (NHANES) population.

	Hypothyroidism OR (95% CI)	P value
Unadjusted	1.29 (0.81–2.06)	0.255
+Age	0.94 (0.54–1.66)	0.830
+Sex	1.02 (0.56–1.86)	0.948
+Race	1.09 (0.61–1.96)	0.757
+Comorbidities	0.98 (0.57–1.67)	0.925
+SES	1.06 (0.64–1.77)	0.797
+HRB	1.07 (0.62–1.85)	0.797
+General health conditions	1.05 (0.59–1.88)	0.875

SES: Socioeconomic status variables included annual household income and education level.

HRB: Health related behaviors include smoking status (current, past, or never), and alcohol intake (current, past, or never).

Comorbidities include diabetes, BMI.

Table 5 shows the overall demographic, comorbidity, and health-related behavior information for the NHIS dataset. The mean age was 46 years, and the racial profile of NHIS was comprised of 69.1% non-Hispanic white, 13.58% Hispanic, 11.8% non-Hispanic black, 4.6% non-Hispanic Asian, and 0.87% other.

**Table 5:** Overall Demographics for the National Health Interview Survey (NHIS).

	Mean	Standard Deviation
Age (years)	45.96	17.7
Gender, female %	51.71	
Race, %		
Hispanic	13.58	
Non-Hispanic White	69.14	
Non-Hispanic Black	11.77	
Non-Hispanic Asian	4.63	
Other	0.87	
Education, %		
<9^th^ Grade	5.70	
> = 9^th^ Grade, <HS graduate	9.74	
HS graduate or GED equivalent	27.70	
Some college	20.22	
College graduate and byeond	36.63	
Income, %		
<$20,000	30.68	
$20,000-$44,999	36.64	
$45,000-$74,999	20.13	
$75,000 and up	12.55	
Glaucoma, %	2.25	
Hypothyroidism, %	6.93	
BMI	30.49	15.5
Diabetes, %	8.28	
High cholesterol, %	32.63	
Hypertension, %	29.46	
Heart attack, %	3.62	
Migrane, %	18.05	
Stroke, %	2.87	
Smoking, %		
Current	20.65	
Former	21.58	
Never	57.77	
Alcohol, %		
Current	64.44	
Former	14.52	
Never	21.04	
General health condition, %		
Excellent-Good	86.87	
Fair	9.84	
Poor	3.29	

Table 6 shows the overall demographic, comorbidity, and health-related behavior information for the NHANES dataset. The mean age was 36.5 years in NHANES, and the racial profile of NHANES was comprised of 66.3% non-Hispanic white, 9.9% Mexican, 5.4% other Hispanic, 12.2% non-Hispanic black, and 6.2% other or multiracial.

**Table 6:** Overall Demographics for the National Health and Nutrition Examination Survey (NHANES).

	Mean	Standard Deviation
Age (years)	36.49	22.09
Gender, female %	51.03	
Race, %		
Mexican	9.88	
Non-Hispanic White	66.26	
Non-Hispanic Black	12.23	
Other Hispanic	5.39	
Other Multiracial	6.24	
Education, %		
<9th Grade	6.86	
> = 9th Grade, <HS graduate	13.65	
HS graduate or GED equivalent	25.48	
Some college	2.88	
College graduate and beyond	25.21	
Income, %		
<$20,000	1.61	
$20,000-$44,999	26.81	
$45,000-$74,999	21.57	
$75,000 and up	31.81	
>$20,000	3.71	
Glaucoma, %	4.71	
Hypothyroidism, %	9.77	
BMI	26.43	7.30
Diabetes, %		
Yes	6.41	
Borderline	1.18	
Heart attack, %	3.30	
Stroke, %	3.17	
Smoking, %		
Current	22.79	
Former	24.11	
Never	53.10	
Alcohol, %		
0	36.54	
>0 and <1	47.03	
≧1 and <2	9.33	
≥2 and <4	5.23	
≥4	1.87	
General health condition, %		
Excellent-Good	45.54	
Fair	38.48	
Poor	15.98	

There were 3,752 participants included in the ancillary analysis, which included laboratory information on thyroid tests and also noted prescription of medications for hypothyroidism. Among this population, 243 self-reported a diagnosis of glaucoma and 661 were diagnosed as having hypothyroidism, representing 4.7% and 16.5% of the sample population, respectively. Table 7 summarizes the characteristics of participants with and without laboratory-confirmed hypothyroidism. When the hypothyroidism patients were further divided based upon whether or not they had received a prescription for levothyroxine, 365 were classified as having untreated hypothyroidism and 296 as being treated hypothyroid patients. The unadjusted and adjusted odds ratios for glaucoma amongst hypothyroidism were 1.17 (95% CI 0.84–1.63) and 0.79 (95% CI 0.52–1.21), respectively. When the hypothyroidism group was further divided into two groups according to the need for thyroid replacement therapy, the unadjusted and adjusted ORs for the untreated hypothyroidism group were 0.83 (95% CI 0.52–1.31), and 0.50 (95% CI 0.23–1.09), respectively (Table 8). Likewise, unadjusted and adjusted ORs for the treated hypothyroidism group were 1.50 (95% CI 0.93–2.41) and 1.07 (95% CI 0.60–1.91), respectively. None of the unadjusted or adjusted ORs were statistically significant in the NHANES sample. Table 9 shows the proportions of participants with and without self-reported hypothyroidism in three age groups in order to provide a better understanding of the effect of age in our analysis.

**Table 7:** Demographics for National Health and Nutrition Examination Survey (NHANES) based on laboratory hypothyroidism

	No Hypothyroidism (N = 3091)		Hypothyroidism (N = 661)		
	Mean	Standard Deviation	Mean	Standard Deviation	P value
Age (years)	56.39	10.62	60.07	12.75	<0.001
Gender,female %	48.49		71.6		<0.001
Race, %					0.3109
Mexican	5.83		5.14		
Other Hispanic	4.30		3.08		
Non-Hispanic white	74.21		74.13		
Non-Hispanic black	10.02		12.44		
Other and multiracial	5.65		5.21		
Education, %					0.5566
<9th Grade	8.35		8.18		
> = 9th Grade, <HS graduate	12.65		13.61		
HS graduate or GED equivalent	25.92		27.89		
Some college	26.35		26.94		
College graduate and beyond	26.73		23.38		
Income, %					0.1964
<$20,000	14.73		18.26		
$20,000-$44,999	27.00		29.83		
$45,000-$74,999	20.34		18.97		
$75,000 and up	34.26		28.85		
>$20,000	3.66		4.09		
Glaucoma, %	4.62		5.35		0.3309
Diabetes, %					0.0041
Yes	11.41		17.21		
Borderline	2.15		1.16		
Hypertension, %	40.75		49.13		0.0015
Stroke, %	4.05		7.77		0.0005
BMI	28.78	5.71	29.75	6.81	0.0006
Smoking, %					0.3842
Never	49.92		53.97		
Current	19.40		18.72		
Past	30.68		27.31		
Alcohol, %					0.0435
0	41.6		51.63		
>0 and <1	43.41		38.66		
≧1 and <2	7.85		5.69		
≧2 and <3	5.11		2.92		
≧4	2.03		1.09		
Genral Health Condition, %					0.0114
Excellent-Good	42.64		32.74		
Fair	38.54		39.17		
Poor	18.82		28.09		

All means, proportions, and standard erros are weighted estimates of the U.S. population characteristics, taking into account NHIS’s complex sampling design.

**Table 8:** Odds ratios (Ors) of the hypothyroidism by laboratory data for self-reported glaucoma for National Health and Nutrition Examination Survey (NHANES) population.

	Hypothyroidism OR (95% CI)	P value
Unadjusted	1.17 (0.84–1.63)	0.331
+Age	0.87 (0.55–1.39)	0.548
+Sex	0.92 (0.57–1.46)	0.694
+Race	0.91 (0.58–1.45)	0.683
+Comorbidities	0.90 (0.58–1.38)	0.600
+SES	0.89 (0.61–1.29)	0.509
+HRB	0.81 (0.54–1.21)	0.279
+General health conditions	0.79 (0.51–1.24)	0.286

SES: Socioeconomic status variables included annual household income and education level.

HRB: Health related behaviors include smoking status (current, past, or never), and alcohol intake (current, past, or never).

Comorbidities include diabetes, BMI.

**Table 9:** Proportions of participants with glaucoma in different age groups separated by presence of self-reported hypothyroidism.

	NHIS		NHANES	
	No Hypothyroidism	Hypothyroidism	No Hypothyroidism	Hypothyroidism
Glaucoma Among participants Age 40–60 years, %	1.24	2.53	2.32	3.16
Glaucoma among participants age 60–80 years, %	5.50	5.78	7.16	7.03
Glaucoma among participants age >80 years, %	12.16	9.37	15.27	11.15

## Discussion

This study, based on two U.S. population samples, the 2008 NHIS and NHANES for 2007 and 2008, showed no statistically significant association between self-reported hypothyroidism, self-reported thyroid disease, or laboratory confirmed hypothyroidism and self-reported glaucoma, after adjustment for potential confounders such as age, gender, race, socioeconomic factors, and other health conditions.

A previous study based on NHANES III (1988–94) found that the prevalence of hypothyroidism, defined by a TSH level >4.5 mIU/liter, was 4.6% in the U.S. population at least 12 years of age.[20] Our findings of a hypothyroidism prevalence of 16.5% in the 2007–2008 NHANES population was substantially higher than that reported for NHANES III but it is noteworthy that we only included subjects over the age of 40, rather than using a 12 year age cutoff in our analysis.

There are several previous population-based studies that assessed the association between thyroid disease and OAG amongst the first of which was The Blue Mountains Eye study (BMES).[12] This study, which was conducted in a primarily Caucasian Australian population, revealed that thyroxin use at the time of the study and past thyroid surgery were significantly associated with an examination confirmed diagnosis of OAG. The thyroid condition of study subjects was ascertained only by the questionnaire with no laboratory confirmation as was used in one cohort of our study.

Motsko et al.[13] reported the lack of an association between hypothyroidism and OAG in a study population derived from a U.S. managed care database including those 60 years of age or older. The diagnoses of OAG, hypothyroidism, and other comorbidities were obtained from the diagnostic coding in the database, and newly diagnosed OAG patients were compared to age- and gender-matched control subjects. Although the primary outcome did not reach statistical significance in the entire study group, an analysis employing the division of hypothyroidism patients into two groups based upon the use of thyroid hormone replacement therapy suggested a possible, protective effect of such treatment.

Lin et al.[15] reported a significantly greater risk of OAG development among hypothyroid patients using the national registry from Taiwan. This study included individuals 60 years or older and reported hypothyroidism patients had a significantly higher risk of OAG after the 5-year follow-up period compared to the comparator group comprised of those who didn’t have hypothyroidism. One must consider, however, that this study only included ethnic Chinese individuals and thus comparisons with populations in other countries may not be appropriate.

Cross et al.[14] reported a positive association between thyroid disease and glaucoma based on analysis of the 2002 NHIS population database. One of the limitations of their study was that results were based only on the answers to a questionnaire that asked whether or not participants had a “thyroid problem,” not distinguishing between hyper- and hypothyroidism. One cannot rule out the possibility that their findings were primarily attributable to a possible link between hyperthyroidism and OAG. It is noteworthy that the NHANES portion of our study also asked whether or not subjects had “thyroid disease,” similar to the question used in the 2002 NHIS.

Kim et al.[16] reported a positive association between OAG and thyroid disease based on their population-based study from South Korea. This study that included people aged 40 years or older has shown that thyroid disease was significantly associated with the diagnosis of OAG in their multivariate model. In this study, the diagnosis of thyroid condition was also obtained through their questionnaire and didn’t distinguish hyperthyroidism from hypothyroidism.

Calissendorff et al.[21] reported a lack of an association between hypothyroidism and the risk of developing open-angle glaucoma in a Swedish population of over 2 million individuals aged 60–89 years. The study was based on the database that has nationwide coverage of all filled prescriptions and assessed whether or not there was an association between thyroxin substitution and anti-glaucoma medication prescription. A significant limitation of their study was the assumption that those with glaucoma and hypothyroidism were not under diagnosed using this surrogate measure of prescribed medications.

In the NHANES population, we were able to perform a sub-analysis based on information obtained from laboratory measures of thyroid hormone and from medication usage. The results failed to show a significant association between the self-reported diagnoses of glaucoma and thyroid disease, or hypothyroidism diagnosed by laboratory data and prescription information.

As the underlying hypothesis for the increased risk of having glaucoma among hypothyroidism patients is increased IOP due to low metabolism caused by the hypothyroid state, several studies have looked at whether or not taking thyroid hormone replacement is beneficial in reducing the risk of developing glaucoma. For example, the BMES [12] reported that levothyroxine use is a risk factor for the development of OAG while, in contrast, Lin et al.[15] reported that taking levothyroxine seemed to possibly be protective against the disease although the latter group’s finding was not statistically significant.

Our study has several limitations. First the data derived from the NHIS are all based on self-reported information and may be affected by participant recall bias. Thus, there is a chance that we may have underestimated the proportion of subjects who have glaucoma or hypothyroidism in this population. Patty et al.[22] reported the validity of self-reported eye disease based on the Los Angeles Latino Eye Study population; the sensitivity and specificity of self-reported glaucoma among the Latino population were 37.7% and 96.3%, respectively. Since their study is based on the Latino population, the result may not to be directly applicable to other datasets such as ours, which surveyed all ethnicities in the United States. In addition, previous studies have shown discrepancies between self-reported thyroid disease and thyroid disease by medical report. In one nationwide study in Denmark, the sensitivity of the self-reported diagnosis of either hypothyroidism or hyperthyroidism was 0.98, whereas the specificity was 0.57 and 0.67 for self-reported hyperthyroidism and hypothyroidism, respectively.[23] Our results should be interpreted cautiously since self-reported diagnoses may underestimate the number of Americans with glaucoma and/or hypothyroidism. In the NHIS population we divided the participants into two groups based on their response to whether or not they had a hypothyroid condition. However, there was no way to exclude the participants with a hyperthyroid condition in the control group, and that may have contributed to the higher proportion of self-reported glaucoma in this group, thus potentially masking a real association between self-reported glaucoma and self-reported hypothyroidism. Meanwhile, the questionnaire used in the NHANES 2007–2008 asked whether or not participants had “thyroid disease” without specifically ascertaining whether they suffered from hyper- or hypothyroidism. However, our sub-analysis that examined laboratory results for thyroid hormone and levothyroxine use to determine hypothyroidism did not find a significant association between self-reported glaucoma and the diagnosis of hypothyroidism. Detailed information regarding the severity of glaucoma and hypothyroidism, or information regarding subcategories of these two diseases, was not available in the NHIS or the NHANES, which is another potential limitation of our analysis.

In summary, this study, based on two US nationwide surveys, failed to confirm previously reported associations between hypothyroidism and glaucoma. Prospective longitudinal studies are needed to assess whether or not hypothyroidism is truly a risk factor for development and progression of glaucoma.
